# Facebook addictions in low and high schizotypal individuals

**DOI:** 10.1192/j.eurpsy.2023.618

**Published:** 2023-07-19

**Authors:** Y. Boukadida, F. Fekih-romdhane, R. Away, M. Stambouli, W. Cherif, M. Cheour

**Affiliations:** Department of Psychiatry Ibn Omrane, razi hospital, tunis, Tunisia

## Abstract

**Introduction:**

Over the past decades, the digital landscape has been rapidly changing worldwide, and the number of social media users has been constantly increasing. social media usage is widespread among the age groups going from Adolescents (aged 16-24 years) to young adults (aged 25-34 years) the vast majority of Tunisian students (98.4%) reported using social media platforms, with Facebook being the most widely used (94.3%), followed by YouTube (90.5%) and Instagram (65.0%) (Feten Fekih-Romdhane et al., 2021)

**Objectives:**

We aimed to compare Facebook addiction between low and high schizotypal individuals

**Methods:**

the final sample included in this study was comprised of 700 students. Based on the Schizotypal Personality Questionnaire (SPQ) total scores, the sample was classified into two groups of low (the lower 10% of a standardization sample) and high (the upper 10%) schizotypy(Raine, 1991).

**Results:**

From the overall pool of 700 students (67.6% females, mean age of 21.5 ± 2.5 years), 74 identified as belonging to the low schizotypy group and 70 were classified as having high schizotypal traits .

Students of the high schizotypy group displayed significantly greater scores on Facebook (p=.001) addiction scales, as compared to those of the low schizotypy group

High schizotypal participants spent in average 7.4 hours on on social media use per day, as compared 3.9 hours on social media in their low schizotypal counterparts

**Conclusions:**

Our study showcases that schizotypy is related to a certain level of addiction to social media. These results can be used as a way to help this at risk population by making social media into a safe space where we can provide help lines and protection

**Disclosure of Interest:**

None Declared

